# Case Report: Recurrence of Testicular Myofibroblastic Tumor After Surgery

**DOI:** 10.3389/fonc.2021.810708

**Published:** 2022-01-14

**Authors:** Jiayi Liu, Zhijie Bai, Shuaiqi Li, Sheng Zeng, Chuang Li, Qian Liu

**Affiliations:** ^1^ The First Central Clinical College of Tianjin Medical University, Tianjin, China; ^2^ Department of Urology, Tianjin First Central Hospital, School of Medicine, NanKai University, Tianjin, China; ^3^ School of Medicine, Nankai University, Tianjin, China

**Keywords:** case report, testis, spindle cell neoplasms, inflammatory myofibroblastic tumor, plasma cell granuloma, inflammatory pseudotumour, recurrence

## Abstract

Inflammatory myofibroblastic tumour (IMT), also known as plasma cell granuloma (PCG) or inflammatory pseudotumour (IPT), is a distinctive, rarely metastasizing neoplasm composed of myofibroblastic and fibroblastic spindle cells accompanied by inflammatory infiltration of plasma cells, lymphocytes and/or eosinophils. IMT predominantly affects children and young adults, and the age at presentation ranges from 3 to 89 years. We present a very rare case of recurrent testicular IMT without ALK rearrangement. This case highlights the clinical characteristics and diagnostic factors associated with primary and recurrent foci of this rare tumour, along with key therapeutic approaches.

## Introduction

Inflammatory myofibroblastic tumour (IMT), also known as plasma cell granuloma (PCG) or inflammatory pseudotumour (IPT), is a distinctive, rarely metastasizing neoplasm composed of myofibroblastic and fibroblastic spindle cells accompanied by inflammatory infiltration of plasma cells, lymphocytes and/or eosinophils (1, 2). IMT predominantly affects children and young adults, and the age at presentation ranges from 3 to 89 years (3). IMT has been reported in multiple locations but is typically located in the lung, liver, biliary tract, or gastrointestinal tract (4). IMT is particularly rare in the urogenital system but usually involves the bladder and the kidney when occurring in this location (5, 6). We report the first case of a patient with recurrent testicular IMT and review the relevant literature to raise awareness of the clinical characteristics and diagnostic factors associated with primary and recurrent foci of this rare tumour, along with key therapeutic approaches.

## Case Presentation

**Figure 1:**
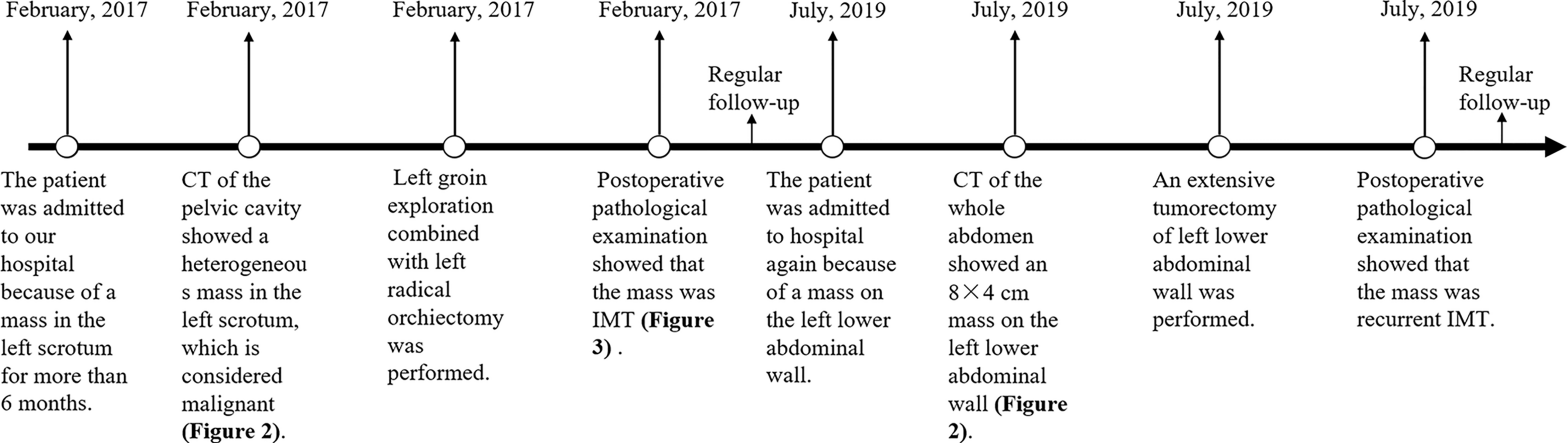
Time axis. Time axis of treatment of primary and recurrent tumours.

The patient was a 58-year-old man who was admitted to our hospital for mass in the left scrotum that had persisted for over 6 months, accompanied by scrotal pain and discomfort and frequent, urgent and incomplete urination. Nocturia occurred 3–4 times per night without painful urination. The patient did not experience gross haematuria, fever, back and abdominal pain, loss of appetite or weight loss. Diagnosis and treatment of the patient is shown in **Figure 1**. Medically, he was healthy and had no known drug allergies. He was a non-smoker and reported no alcohol consumption. On physical examination, a tough, smooth mass approximately 10×8 cm in size was palpable in the left scrotum with mild tenderness. The light transmission test results were negative, and thickening of the left spermatic cord was observed. The external genitalia and the right testicular epididymis were normal.

**Figure 2:**
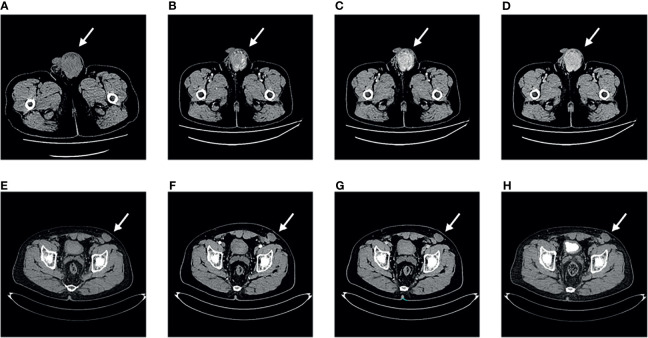
Computed tomography findings. Pelvic computed tomography of primary tumour **(A–D)**. **(A)** Plain scan. **(B)** Arterial phase. **(C)** Venous phase. **(D)** Excretion phase. Pelvic computed tomography of recurrent tumour **(E–H)**. **(E)** Plain scan. **(F)** Arterial phase. **(G)** Venous phase. **(H)** Excretion phase.

Routine blood test results, blood biochemistry analyses, tumour marker levels and hormone levels (alpha-fetoprotein, AFP; carcinoembryonic antigen, CEA; glycoprotein antigen 199, CA199; glycoprotein antigen 724, CA724; keratin 19 fragment, CYFRA; neuron enolase, NSE; beta subunit of human chorionic gonadotropin hormones, β-HCG; testosterone, TESTO; prolactin, PRL; progesterone, PROG; luteinizing hormone, LH; follicle stimulating hormone, FSH and estradiol, E2) were normal. CT of the pelvic cavity showed a heterogeneous mass in the left scrotum, which was considered malignant because the mass is obviously enhanced in the arterial phase (**Figure 2**).

**Figure 3:**
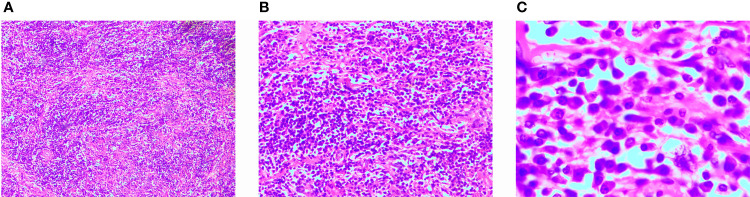
Histological findings. Hematoxylin and Eosin staining of paraffin-embedded sections. **(A)** Magnification × 40. **(B)** Magnification × 100. **(C)** Magnification × 400.

Informed consent was obtained from the patient to perform left groin exploration with left radical orchiectomy. A round, smooth and hard mass with a clear boundary measuring approximately 10×8 cm was observed during the surgery. The left spermatic cord was ligated and the testis was removed. The postoperative pathological examination revealed abnormal testis parenchymal structure with large numbers of infiltrating lymphocytes and plasma cells, lymphatic follicles, and proliferating fibroblasts and myofibroblasts; additionally, scattered R-S cells could be seen in some areas (**Figure 3**). The postoperative immunohistochemical examination revealed the following: S100 (scattered +), Actin (-), ALK (-), CD34 (-), CD68 (+), IgG (plasma cell +), Kappa (+), Lambda (+), Ki-67 (10%), CD20 (corresponding +), CD138 (plasma cell +), CK (-), CD3 (corresponding +) and IgG4 (10%+). A postoperative diagnosis of testicular IMT was made based on the examination findings.

The patient attended follow-up appointments once a year after surgery. At the 27th month of follow-up, the patient presented with a mass in the left lower abdominal wall. No symptoms of fever, cough, night sweats, or abdominal pain were present, and no significant change in body weight was observed. B-ultrasonography revealed a deep subcutaneous mass in the left lower abdominal wall. The patient returned during the 29^th^ month of follow-up due to the slow and painless growth of the mass in the left lower abdominal wall. A physical examination revealed that the mass was immobile, approximately 4×3 cm in size and tough in quality. B-ultrasonography indicated a hypoechoic solid mass in the proximal area of the left lower abdominal wall, approximately 4.3×3.1×2.5 cm in size. The boundary was unclear and irregular, and the echo was not uniform. CDFI revealed low blood-flow signals. Enhanced pelvic CT examination also showed the same result; thus, the tumour was considered to be *in situ* IMT (**Figure 2**). Informed consent was obtained from the patient to perform an extensive tumorectomy of the left lower abdominal wall. During the surgery, it was found that the tough nodular mass had infiltrated and grown into the subcutaneous tissue, involving the obliquus externus abdominis and the obliquus internus abdominis, covering an area of approximately 4×3 cm. Thus, the surgeon expanded the excision along the edge of the tumour to remove the complete specimen. The base of the tumour extended outside of the peritoneum. Postoperative pathological analyses revealed fibrous tissue hyperplasia with extensive lymphocyte and plasma cell infiltration, consistent with IMT. Postoperative immunohistochemistry showed the following: ALK (-), desmin (-), SMA (-), CD163 (histocyte +), lysozyme (-), CD30 (-), CD21 (-) and CD35 (-). The patient was diagnosed with recurrent IMT based on the results of these postoperative examinations. The patient attended follow-up for another 27 months, and no further recurrence occurred.

## Discussion

IMT, also known as PCG or IPT, is a distinctive, rarely metastasizing neoplasm composed of myofibroblastic and fibroblastic spindle cells accompanied by inflammatory infiltration of plasma cells, lymphocytes and/or eosinophils (1, 2). IMT prevailing affects adolescents, and the age at presentation ranges from 3 to 89 years (3). IMT has been reported in multiple locations but is typically located in the lung, liver, biliary tract, or gastrointestinal tract (4). IMT rarely occurs in the urinary system (5, 6). To the best of our knowledge, only six patients with intratesticular IMT have been reported previously, none of whom experienced recurrence or metastasis (7–12) (**Table 1**). We describe the first case of a patient with intratesticular IMT recurrence.

**Table 1:** Previously reported patients with intratesticular IMT.

Author	Year	Age	Gender	Presentation	Quantity	Diameter/Size(cm)	Diagnostics	Therapy	ALK status	Recurrence or metastasis
Aksoy PK (7)	2001	45	Male	a painless right testics mass	1	3.5×2.0×2.0	Postoperative pathology and immunohistochemistry	Right radical orchiectomy	NS	NS
Navai N (8)	2005	30	Male	a painless right testics mass	1	1.8×1.5×1.0	Postoperative pathology and immunohistochemistry	Right radical orchiectomy	NS	No
Nistal M (9)	2011	33	Male	a painless right testics mass	1	0.9	Postoperative pathology and immunohistochemistry	Right radical orchiectomy	+	No
Torres Gómez FJ (10)	2012	69	Male	a painful right testics mass	1	3.0	Postoperative pathology and immunohistochemistry	Right radical orchiectomy	NS	NS
Hickman RA (11)	2016	22	Male	a painless right testics mass	2	1.0cm and 0.9	Postoperative pathology and immunohistochemistry	Right radical orchiectomy	+	NS
Voelker HU (12)	2017	40	Male	a painless left testics mass	1	3.0	Postoperative pathology and immunohistochemistry	Left radical orchiectomy	–	No

NS, not stated.

The aetiology of IMT remains unclear. Common aetiological factors of IMT are trauma, surgery, inflammation, and abnormal repair (13). Infections with Epstein-Barr virus, specific bacteria, and human herpesvirus have been considered to play an important role in the pathogenesis of pulmonary IMT (14, 15). However, Wei Song and Yan Zhu determined that IMT is not associated with Epstein-Barr virus infection by analyzing the clinical characteristics and outcomes of 17 patients with IMT at a university hospital in China (16). Therefore, the specific aetiology of IMT needs further study.

Patients with IMT of the lung or abdomen usually present with masses or nonspecific symptoms, including abdominal pain or gastrointestinal discomfort caused by abdominal lesions and cough, chest pain, or in rare cases, haemoptysis (17). In some cases, masses are found only after extensive investigation into unexplained fevers or growth disorders. The clinical manifestations of testicular IMT are similar to those of other painless testicular tumours (18). Of the six previously reported cases and this case, 5 (71.4%) patients presented with painless masses, and 2 (28.6%) presented with painful masses. A very small number of patients with testicular tumours have rapid-onset symptoms, such as sudden painful masses, local redness and swelling with fever, mostly caused by tumour haemorrhage, infarction, and necrosis; however, sudden symptoms have not been reported in testicular IMT patients. It is difficult to distinguish testicular IMT from other testicular tumours due to the similarities in the clinical manifestations.

Serum tumour markers are critical to distinguish testicular IMT from germ cell tumours. α-Fetoprotein (AFP), human chorionic gonadotropin (hCG), lactate dehydrogenase (LDH), and placental alkaline phosphatase (PLAP) are generally and significantly elevated in patients with testicular germ cell tumours (19). Correspondingly, the levels of the abovementioned tumour markers were normal in the 7 reported cases of IMT. Serum IL-6 levels were reported to be elevated in two IMT patients and returned to normal after surgery as systemic symptoms subsided, and several authors have demonstrated that tumour cells produce IL-6 mRNA and proteins, indicating a potential mechanism underlying IMT (20–22). However, serum IL-6 was not detected in the 7 reported cases of testicular IMT. Due to the low morbidity level of the disease, information about serum tumour markers of testicular IMT is lacking, and these markers require further study.

B-ultrasound, CT, and magnetic resonance imaging are commonly used imaging examinations to diagnose scrotal masses. Although imaging examinations play an important role in diagnosing IMT, postoperative histological examination is the gold standard (23, 24). The imaging manifestations of IMT are mass-like lesions with or without patches eroding the surrounding tissue. Space-occupying expanded soft tissue can present with different densities on imaging, suggesting mixed tissue types. IMT masses may be rich in blood vessels and adhesions, and compression and damage to the local area may occur, similar to that observed with other malignant tumours. US of IMT typically shows solid mixed tissue echoes with well-defined boundaries or infiltration. Doppler ultrasonography typically shows significant vascular formation. Plain CT usually shows nonuniform signal attenuation, and a few lesions involve the surrounding area. Plain CT also typically shows different types of intravenous contrast agent enhancement, including no enhancement, nonuniform enhancement and peripheral enhancement. If lesions are large, plain CT may show low attenuation in the central area, suggesting necrosis (25). Calcification is rare in IMT and usually occurs in adult head and neck IMT (26). IMT lesions show persistent enhancement. Most IMT lesions are soft tissue masses with isointense to hypointense signals on T1W imaging and heterogeneously high signals on T2W imaging, depending on the cell contents, and contrast-enhanced MR imaging shows variable enhancement patterns (26). The uptake of FDG by IMTs varies from low to high, which may be due to the structure of the tumour cells, the biological behaviour of the tumour cells, the composition and proportion of inflammatory cells in the tumours, and the degree of activation of those inflammatory cells (14). FDG positron emission tomography (PET)/CT may be useful for detecting primary tumours, local recurrences, and distant metastases. Because IMT has no specific imaging characteristics, imaging cannot support clinicians in making an accurate preoperative diagnoses. The preoperative imaging examinations of all 7 cases of reported testicular IMT were unclear.

The definitive diagnosis of IMT is dependent on postoperative pathological and immunohistochemical examinations. Histologically, IMT is characterized by varying proliferation of spindle cells in a myxoid to a collagenous matrix, and a significant inflammatory infiltrate consisting mainly of plasma cells and lymphocytes with occasional eosinophils and neutrophil involvement (13). Three types of IMT tissue can be identified microscopically. In the spindle cell type, the tumour cells are arranged in a zigzag or spiral pattern, surrounded by newly formed plasma cells of vascular integrations, similar to spindle cell tumours such as fibrohistiocytomas, smooth muscle tumours, and gastrointestinal stromal tumours. In the mucinous type, obvious interstitial oedema and mucinous alterations are observed with spindle cells, various inflammatory cells and luteinized tissue cells. In the fibrous type, flaky fibrotic stroma with lymphocytes and numerous plasma cells is observed as a plasma cell granulomatous image that resembling scarring or banded fibromatosis (17).

Immunohistochemically, 90% of IMTs express SMA and 50% express pancytokeratin focally. IMT is typically positive for low-molecular-weight cytokeratin and negative for high molecular weight cytokeratin P63 and epithelial membrane antigen. Approximately 50% of IMTs are positive for desmin (27). Approximately 50% of IMTs have a clonal rearrangement of the ALK gene (2p23, anaplastic lymphoma kinase). ALK-negative IMTs are more often found in elderly patients and exhibit a higher degree of cellular pleomorphism and mitotic activity and a higher risk for local recurrence (17). Both the primary and recurrent IMTs were ALK-negative in our patient, as determined by the immunohistochemical analysis.

Because testicular IMTs are often difficult to distinguish from malignant testicular tumours preoperatively, unilateral radical orchiectomy is generally conducted. A literature review revealed that the first-choice treatment for pathologically confirmed IMT is surgery. IMT is considered to be a benign tumour that does not metastasize. However, if the tumour infiltrates the surrounding tissue, *in situ* relapse can occur despite extended dissection (28). The effect of radiotherapy or chemotherapy after testicular IMT is unknown. Although chemotherapy is recommended for patients with incomplete or impossible resection, it is not a widely accepted treatment modality for testicular IMT. Our patient and all 6 previously reported testicular IMT patients underwent unilateral radical orchiectomy without postoperative chemotherapy, radiotherapy or other treatments. The successful treatment of IMT with anti-inflammatory drugs combined with corticosteroids has also been reported, but this approach has not been used to treat testicular IMT (29, 30). The latest research suggests that clozotinib be used to treat patients with ALK (+) IMT. The pathological analysis of our patient revealed that the testicular and left lower abdominal wall tumours were ALK (-); thus, no further treatment was administered after surgery.

The prognosis of IMT patients is mostly good, and recurrence or metastasis is not common. IMT may recur many years later, even following radical surgery. The incidence of local recurrence is between 15 and 40% (31). Serkan Karaisli, Erdinc Kamer et al. collected follow-up information of 49 colorectal IMT patients, of which 7 experienced relapse without metastasis (32). The recurrence rate is related to the size of the tumours and the abdominal location and is higher among elderly patients (3).

Because of the risk for IMT recurrence or metastasis, our patient received regular follow-up evaluations. At the 27th month of follow-up, our patient presented with a mass in the left lower abdominal wall, which was considered to be recurrent IMT. At the 29th month of follow-up, an extensive tumorectomy of the left lower abdominal wall was performed. Postoperative pathology and immunohistochemistry confirmed that the tumour was recurrent IMT. Our case is the first case of recurrent testicular IMT.

The optimal treatment option for recurrent disease is unclear. A literature review revealed that secondary surgery is the best option when possible, similar to the approach for primary IMT. Nicholas D. Andersen, Louis R. DiBernardo et al. reported the case of a young patient with recurrent cardiac IMT who received secondary surgery after failing to respond to steroid therapy (33). Xinjun Wang, Xiaokun Zhao et al. reported the case of a patient with recurrent retroperitoneal IMT who underwent secondary surgery without postoperative adjuvant therapy (34). Tian Zhang, Yawei Yuan et al. reported the case of a patient with recurrent IMT of the inguinal region who underwent secondary surgery with postoperative fractionated radiotherapy (35). When IMT is encountered, the treatment method should consider factors such as the patient’s general condition, the difficulty of the second operation, the complications that could arise, and the location of the tumour. Our patient continued to receive regular reexaminations after the secondary surgery.

## Conclusion

In conclusion, testicular IMTs are very rare and have only been previously reported in 6 other patients, none of whom experienced recurrence. Because IMT laboratory and imaging characteristics are not specific, making a clear preoperative diagnosis is difficult. The diagnosis of IMT depends on postoperative pathological and immunohistochemical examinations. Primary testicular IMTs are usually treated with radical orchiectomy. The efficacy of chemotherapy, radiotherapy, and anti-inflammatory drugs for testicular IMTs is unclear. Due to the potential for recurrence and metastasis of IMT, patients with testicular IMT should attend regular follow-up appointments, especially patients with ALK-negative testicular IMT. Once recurrence is found, radical tumorectomy should generally be performed, but the general condition of the patient, the difficulty of the second operation, the complications that could arise, and the location of the tumour should also be comprehensively evaluated.

## Data Availability Statement

The original contributions presented in the study are included in the article/supplementary material. Further inquiries can be directed to the corresponding author.

## Ethics Statement

Written informed consent was obtained from the individual(s) for the publication of any potentially identifiable images or data included in this article.

## Author Contributions

JL was involved in the identification, selection, and management of patient cases and wrote and revised the manuscript. SL, ZB, SZ, CL, and QL was involved in the management of patient cases. QL finally reviewed the manuscript. All authors contributed to the article and approved the submitted version.

## Funding

Supported by Tianjin Key Medical Discipline Construction Project; Science and Technology Fund of Tianjin Health and Family Planning Commission (No. 16KG103); Tianjin Health Science and Technology Project (No. ZC20162).

## Conflict of Interest

The authors declare that the research was conducted in the absence of any commercial or financial relationships that could be construed as a potential conflict of interest.

## Publisher’s Note

All claims expressed in this article are solely those of the authors and do not necessarily represent those of their affiliated organizations, or those of the publisher, the editors and the reviewers. Any product that may be evaluated in this article, or claim that may be made by its manufacturer, is not guaranteed or endorsed by the publisher.
